# Audit on Intraductal Papilloma of the Breast: Upgrade Rate, Management Pitfalls, and Updated Guidelines in a Tertiary Health Care Center

**DOI:** 10.7759/cureus.18763

**Published:** 2021-10-13

**Authors:** Hiba Esmayil, Sheraz Abayazeed, Mohamad Hajaj

**Affiliations:** 1 Radiology, Hamad Medical Corporation, Doha, QAT; 2 Breast Surgery, Hamad Medical Corporation, Doha, QAT; 3 Breast Imaging, Hamad Medical Corporation, Doha, QAT

**Keywords:** vacuum-assisted aspiration, vacuum-assisted excision, breast, intraductal papilloma, papillary lesions, b3 lesions, mammogram, biopsy, upgrate rate, dcis

## Abstract

Background

Intraductal papillomas (IDPs) are typically classified as B3 lesions in histology as they may show intralesional heterogeneity with a potential upgrade to malignancy. On core needle biopsy (CNB), a distinction between papilloma versus papillary ductal carcinoma in situ (DCIS) may be difficult. It is well known that otherwise benign papillomas may harbor foci of atypical ductal hyperplasia or DCIS. In this study, we aimed to calculate the radiological (mammogram and ultrasound) accuracy of IDP and to analyze the accuracy of CNB to diagnose IDP. Furthermore, we calculated the percentage of upgrade to malignancy after surgical excision. Any case that had a co-existing in-situ or invasive carcinoma during surgical excision was considered as an “upgrade” to malignancy. Finally, we analyzed the current management protocol for IDP in the institution and suggested changes, if needed.

Methodology

This is a retrospective cross-sectional study. A total of 112 cases diagnosed as IDP radiologically and/or by histopathology over a one-year time frame were included. A retrospective analysis of the accuracy of the radiological diagnosis was done by comparing it with CNB and/or surgical excision biopsy reports. The number of cases diagnosed with a co-existing in-situ or invasive carcinoma was calculated. This was considered as an “upgrade” from a B3 lesion in CNB to carcinoma in surgical excision. Current institutional management protocols were evaluated and compared with international benchmarks.

Results

Out of the 112 cases, 91 were suspected to be papilloma by imaging. The remaining 21 cases who were positive for papilloma on biopsy but were not diagnosed radiologically were also studied separately. Among the biopsied patients, eight were positive for IDP with atypia in CNB. Five out of these eight cases had an in-situ or invasive component during the surgical excision, with one invasive lobular carcinoma, three lobular carcinomas in situ, and one DCIS on surgical excision histopathology. The upgrade percentage was calculated to be 22.72%.

Conclusions

Due to the large upgrade potential of IDP, it is recommended to biopsy every radiologically suspected lesion and excise pathology-proved lesions. If the biopsy shows papilloma without atypia, vacuum excision is sufficient; otherwise, surgical excision with a clearance of margins is advocated. Annual mammograms/surveillance is recommended for biopsy-proven cases. IDP has a high upgrade potential, and, hence, care should be taken to biopsy suspicious lesions. An excision of biopsy-proven lesions must be done.

## Introduction

Intraductal papilloma (IDP) is a benign tumor that comprises approximately 3-6% of breast core biopsy diagnoses. It is commonly classified as a high-risk precursor lesion as there is a possibility of an upgrade to atypical ductal hyperplasia and ductal carcinoma in situ (DCIS) [1].

Clinically, IDP usually presents as a palpable breast mass or unilateral nipple discharge [2]. The mammograms may be usually normal. Patients may present with solitary or multiple dilated ducts, a circumscribed well-defined mass (often retroareolar), or a cluster of calcifications [1]. On ultrasound, the usual findings are of a well-defined solid nodule or dilated duct with an intraductal mass. This may either fill a duct or may even be outlined by fluid partially. Color Doppler ultrasound sometimes demonstrates a vascular stalk. Radiological findings suggestive of papillary breast lesions are usually of low specificity for the diagnosis of malignancy because they cannot definitively distinguish benign lesions from those that are potentially malignant [3].

In this study, any case that had an in-situ or invasive component during surgical excision was considered as an upgrade to malignancy. Previous studies have shown that on core needle biopsy (CNB), IDP without atypia has a 2-7% possibility of upgrade to atypia and carcinoma in surgical excision specimens, whereas papillomas with atypia have a higher upgrade rate of approximately 20%. Therefore, working up an IDP is necessary; however, it is challenging because it displays diverse radiological and pathological features.

CNB may underestimate atypical features due to insufficient sampling. Thus, surgical excision is necessary for patients with atypia or features of malignant transformation seen on biopsy, while the management of IDPs without atypia is still controversial and continues to be managed differently [4,5].

This study aimed to analyze the upgrade rate of IDP to malignancy and the current institutional management protocols, and benchmark the protocols with the National Health Services (NHS) guidelines [6].

## Materials and methods

In this retrospective cross-sectional study, we included patients who presented to our institution from September 2018 to September 2019. Inclusion criteria included patients who were diagnosed with IDP radiologically (via mammogram and ultrasound) and/or histopathologically. We excluded patients who presented with symptoms of nipple discharge but with no ultrasound-identified intraductal mass or histology findings of papilloma. Approval from the ethical committee was not required as per the institutional protocols as this was a retrospective study to analyze the current performance of the institution. Anonymized patient details obtained were charted in a locked excel sheet and a PowerPoint sheet which were accessible only to the authors.

A total of 112 cases were included in the study. The upgrade percentage to malignancy was calculated, and the current institutional management protocols were assessed. Out of these 112 total studied cases, 91 were suspected to be IDP by imaging, and 21 were not suspected to be IDP on imaging but turned out to be IDP in histopathology.

Out of the 91 radiologically suspected cases, only 53 patients in our institution underwent CNB. The rest were lost to follow up. Out of these 53 biopsied patients, 21 were positive for IDP and the remaining 32 were negative for IDP.

## Results

**Figure 1:**
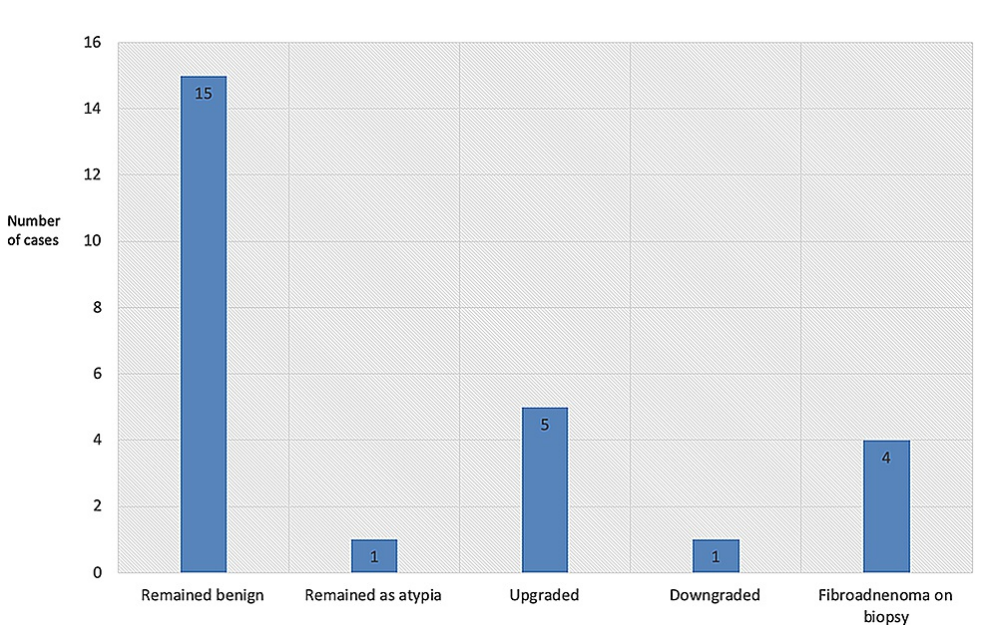
Status of papilloma-proven cases on biopsy that underwent surgical excision.

Among the 42 patients who underwent CNB, eight demonstrated IDP with atypia during the biopsy. As seen in Figure 1, a total of 26 patients (out of the 42 biopsied patients) underwent surgery. Five patients (out of the total eight patients with atypia on biopsy) had an upgrade to invasive lobular carcinoma (one patient), lobular carcinoma in situ (LCIS) (three patients), and DCIS (one patient). Thus, the upgrade percentage in our institution for IDP over one year was calculated to be 22.72%.

One patient who showed features of atypia on CNB demonstrated atypia on excision. Fifteen patients who had benign papilloma on CNB had benign papilloma on excision. One patient was downgraded from papilloma on CNB to ductal hyperplasia. Four patients who had fibroadenoma on CNB turned out to have IDP on excision.

**Figure 2:**
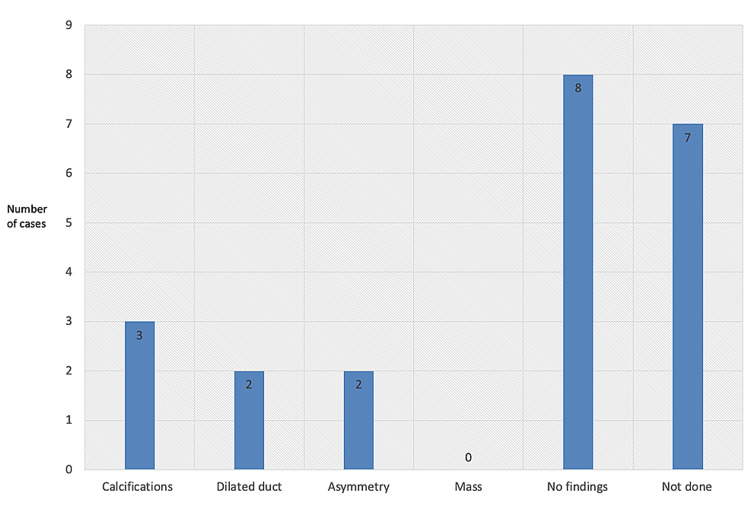
Mammogram findings of the cases that were radiologically suspected.

**Figure 3:**
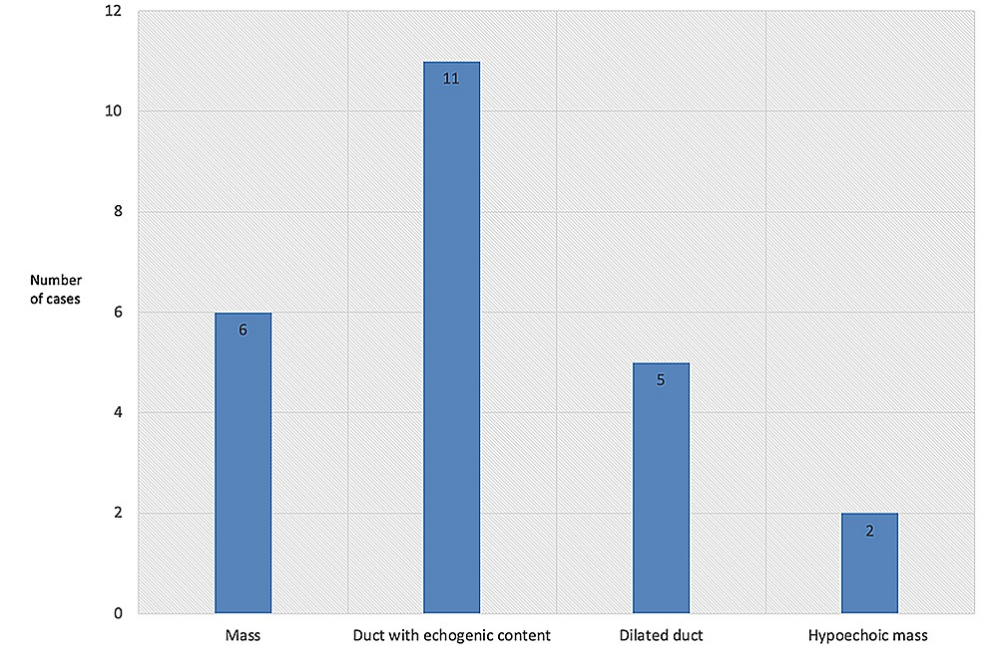
Ultrasound findings of the cases that were radiologically suspected

**Figure 4:**
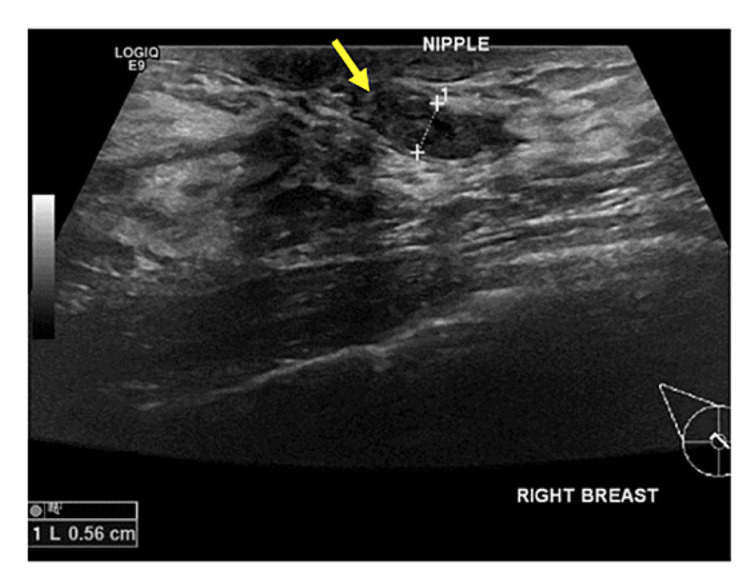
Pathology-proven papilloma with ultrasound findings of dilated retroareolar duct with an echogenic mass within (yellow arrow).

**Figure 5:**
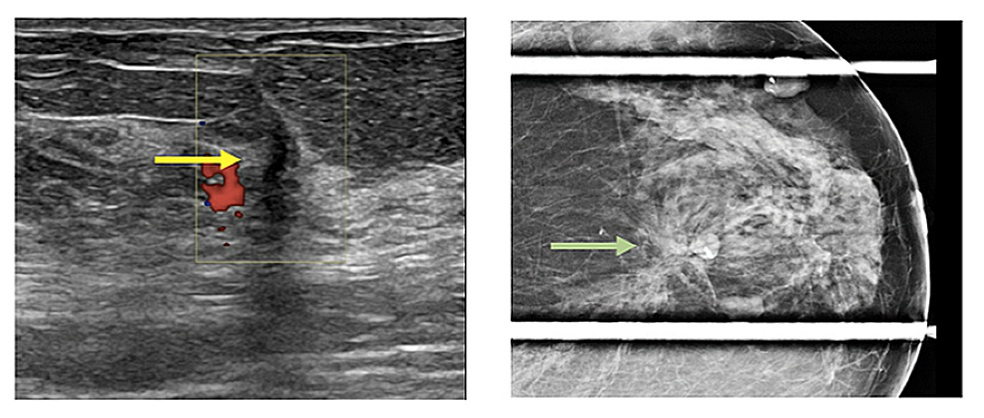
Pathology-proven papilloma with architectural distortion in mammogram (green arrow) and architectural distortion in ultrasound (yellow arrow).

**Figure 6:**
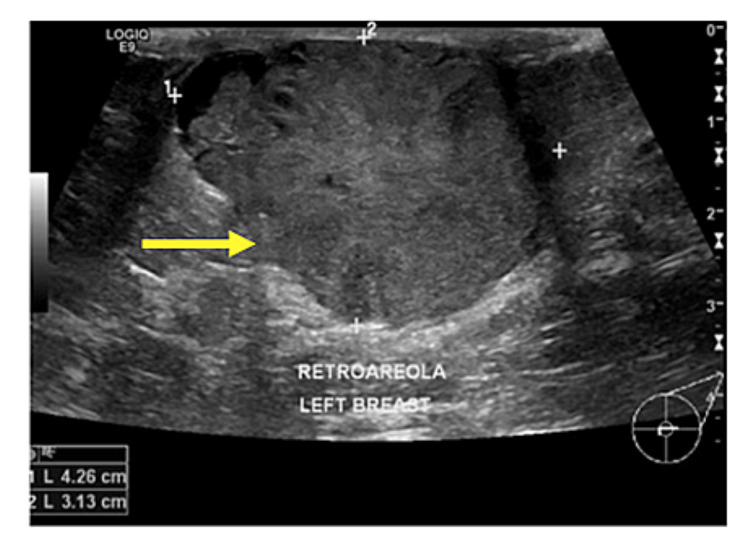
Pathology-proven papilloma presenting as a large retroareolar mass on ultrasound (yellow arrow).

**Figure 7:**
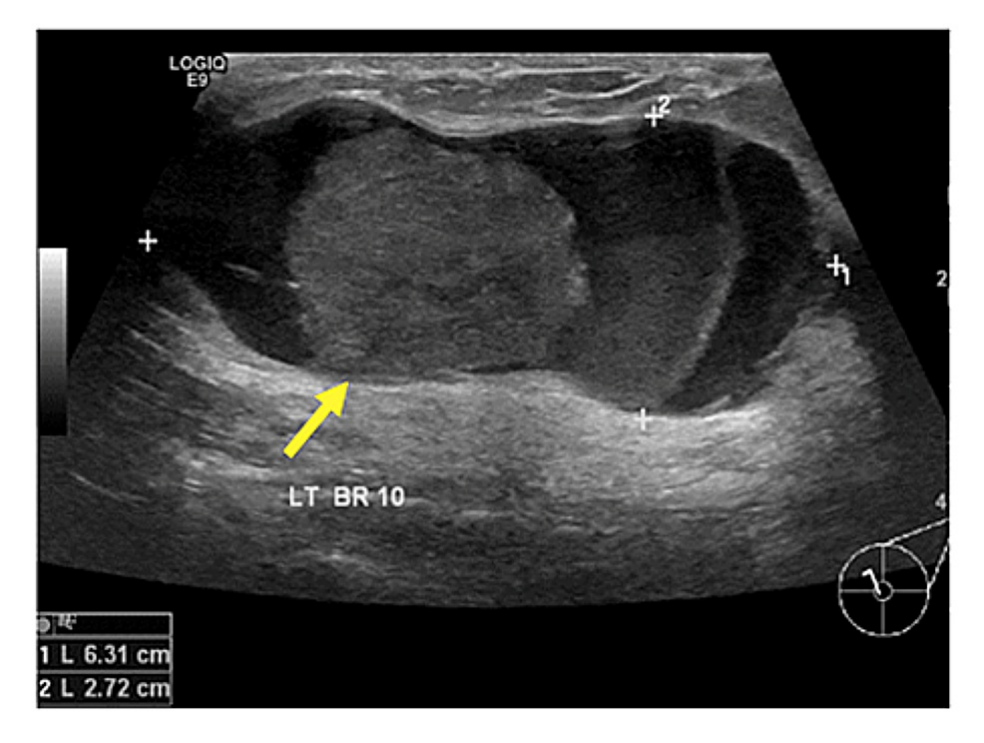
Pathology-proven papilloma with ultrasound finding of a solid lesion within a dilated duct (yellow arrow).

**Figure 8:**
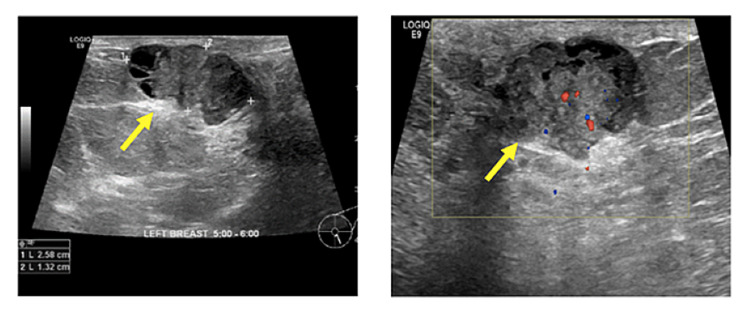
Pathology-proven papilloma with ultrasound findings of a solid lesion with a cystic component (yellow arrow).

Figures 2-8 show the mammogram and ultrasound findings of patients with radiologically suspected IDP. The majority of the patients had no findings on the mammogram (eight out of twenty-one). The most common mammogram finding was calcifications (three out of twenty-one). The rest of the findings on mammograms were dilated duct (two out of twenty-one) and parenchymal asymmetry (two out of twenty-one). The most common finding on ultrasound was echogenic content within the duct (eleven out of twenty-one). The second most common finding on ultrasound was echogenic breast mass (six out of twenty-one). The remaining findings on ultrasound were of dilated duct (five out of twenty-one) and hypoechoic mass (two out of twenty-one). Overall, 52% of the lesions were classified as Breast Imaging Reporting and Data System (BIRADS) III, 33% as BIRADS IVA, and 14% as BIRADS IV.

## Discussion

In summary, 41% of the radiologically suspected cases to be IDP in our institution did not undergo CNB. Overall, 38% of the biopsy-proven IDPs did not undergo excision. Out of the patients who underwent CNB, 12.5% had atypia on CNB and still were not excised. Thus, we made efforts to introduce a new policy for the management of IDP, and all B3 lesions in general, according to the National Health Services (NHS), UK guidelines [6].

A meta-analysis of 34 studies on non-malignant breast papillary lesions by Wen and Cheng included a total of 2,236 non-malignant papillary lesions, among which 346 cases were upgraded to malignant [7]. The pooled underestimation rate was found to be 15.7%, with the underestimation rate for benign IDPs calculated as 7.0% (5.6-8.3%), and the rate for atypical IDPs calculated as 36.9% (29.5-44.3%).

**Figure 9:**
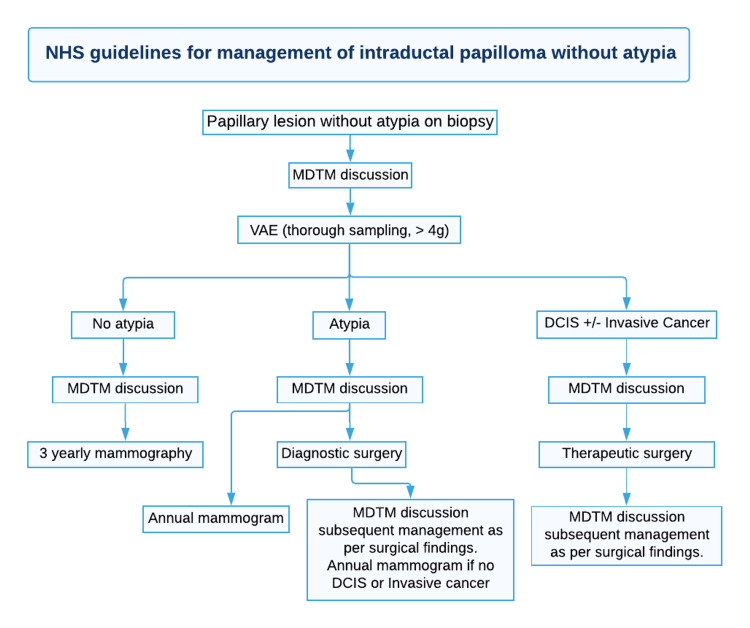
NHS guidelines for the suggested pathway for the management of patients with papillary lesions without atypia in the initial biopsy. NHS: National Health Service; VAE: vacuum-guided excision; MTDM: multidisciplinary team meeting; DCIS: ductal carcinoma in situ

**Figure 10:**
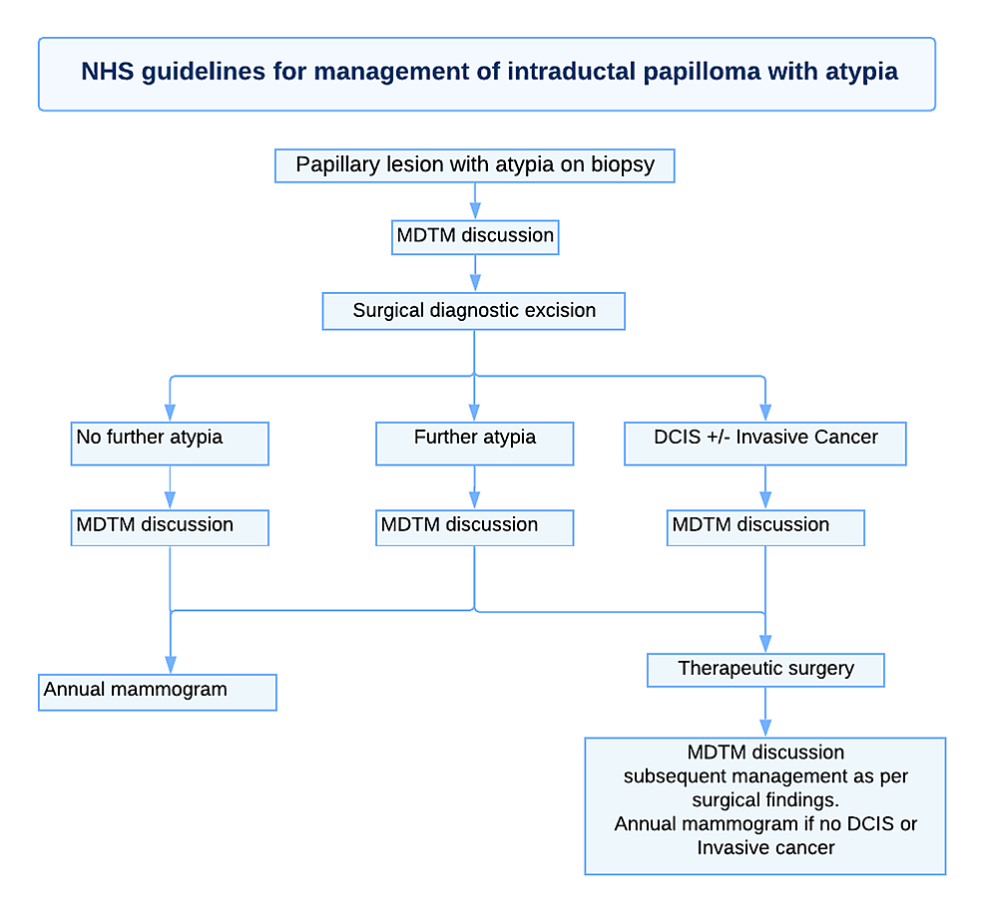
NHS guidelines for the suggested pathway for the management of patients with papillary lesions with atypia in the initial biopsy. NHS: National Health Service; MTDM: multidisciplinary team meeting; DCIS: ductal carcinoma in situ

Given the very high upgrade rates to malignancy for IDP, it is evident that surgical intervention in the form of vacuum-guided excision (VAE) or surgical diagnostic excision is mandatory. Papillary lesions with atypia identified during CNB/vacuum-assisted biopsy (VAB) require an assessment of the extent in the continuity of the atypia, and an examination of the intact specimen with a bigger sample size must be done. Certainly, all cases should be discussed at a multidisciplinary team meeting (MDTM) and dealt with on an individual basis [8]. As seen in Figures 9, 10, for lesions without atypia, VAE is advised, whereas, for lesions with atypia, diagnostic surgical excision is indicated. A 14-G needle was recommended to perform a CNB for all radiologically suspected lesions. If atypical features are not present, complete excision must be performed using VAE. Following this, if no atypical features are seen on excision, discussion on MDTM with three yearly mammograms should be recommended. If atypia is present, diagnostic surgical excision must be done, followed by an MDTM discussion. If there is evidence of DCIS and/or invasive cancer, therapeutic surgical excision, followed by MDTM discussion must be performed [8].

Conversely, if atypical features are present in the CNB samples for a radiologically suspected IDP, diagnostic surgical excision must be performed, followed by an MDTM discussion. If there is evidence of DCIS and/or invasive cancer, therapeutic surgical excision, followed by an MDTM discussion must be performed [8]. 

The aim of this study is to present a review and guide on the management of IDP of the breast. These guidelines, as seen in Figures 9, 10, reflect suggested practice as stated by the NHS breast screening program and approved by the Royal College of Radiology [6]. However, this protocol for surveillance should be kept under review and amended as more data and international guidance become available.

## Conclusions

All radiologically suspected IDPs should undergo CNB with a 14-G needle to provide adequate tissue for analysis. In our study, the upgrade rate of IDP to malignancy was 22.7%. The cases were upgraded as LCIS, lobular carcinoma, and DCIS. Therefore, it is essential to apply a multidisciplinary approach with close communication between the pathologists, the surgeons, and the radiologists when a radiological diagnosis of papilloma is suspected, or when papilloma is diagnosed in core biopsy. This will allow clinicians to plan for a VAB, which, in turn, provides more tissue for a better diagnosis before the therapeutic surgery, thus, preventing the need for a second surgery due to an earlier diagnosis.

It was also evident that imaging features for IDP are variable, and CNB or VAB must be recommended for suspicious lesions. We followed the NHS guidelines for the management of B3 lesions. A recommendation was made to the breast radiologists and surgeons in our institution to excise all biopsy-proven IDPs based on the presence of atypia. Thus, based on data from NHS, we conclude that if the CNB sample shows IDP without atypia, vacuum excision performed by the radiologist to remove the lesion in its entirety is sufficient. This would be a replacement for an excisional surgery and has the benefits of reduced cost, faster procedure with real-time imaging using ultrasound, and better patient healing. Furthermore, if the CNB sample demonstrates atypical features, surgical excision with a clearance of margins is advocated. This would be beneficial as it would lead to an early diagnosis of underlying cancer if any. Annual mammograms/surveillance is recommended for patients with atypical features on CNB/excision, whereas three yearly mammograms are advocated for patients without atypia based on the current NHS guidelines.
